# KML001, a Telomere-Targeting Drug, Sensitizes Glioblastoma Cells to Temozolomide Chemotherapy and Radiotherapy through DNA Damage and Apoptosis

**DOI:** 10.1155/2014/747415

**Published:** 2014-09-10

**Authors:** Seon Rang Woo, Yunhee Ham, Wonyoung Kang, Heekyoung Yang, Sujong Kim, Juyoun Jin, Kyeung Min Joo, Do-Hyun Nam

**Affiliations:** ^1^Department of Neurosurgery, Samsung Medical Center and Sungkyunkwan University School of Medicine, Seoul 135-710, Republic of Korea; ^2^Cancer Stem Cell Research Center, Samsung Medical Center and Sungkyunkwan University School of Medicine, Seoul 135-710, Republic of Korea; ^3^Samsung Advanced Institute for Health Sciences and Technology (SAIHST), Samsung Medical Center and Sungkyunkwan University School of Medicine, Seoul 135-710, Republic of Korea; ^4^Pharmaceutical Division, Komipharm International Co., Ltd., Seoul 429-450, Republic of Korea; ^5^Graduate School of Biomedical Science & Engineering, Hanyang University, Seoul 133-791, Republic of Korea; ^6^Department of Anatomy and Cell Biology, Samsung Medical Center and Sungkyunkwan University School of Medicine, Seoul 135-710, Republic of Korea; ^7^Center for Molecular Medicine, Samsung Biomedical Research Institute, Seoul 135-710, Republic of Korea

## Abstract

Standard treatment for glioblastoma comprises surgical resection, chemotherapy with temozolomide, and radiotherapy. Nevertheless, majority of glioblastoma patients have recurrence from resistance to the cytotoxic conventional therapies. We examined combinational effects of KML001, an arsenic compound targeting telomeres of chromosomes with temozolomide or irradiation, in glioblastoma cell lines and xenograft models, to overcome the therapeutic limitation of chemoradiation therapy for glioblastoma. Although KML001 alone showed little effects on *in vitro* survival of glioblastoma cells, cell death by *in vitro* temozolomide treatment or irradiation was synergistically potentiated by combination with KML001. Since phosphorylated *γ*-H2AX, cleaved casepase-3, and cleaved PARP were dramatically increased by KML001, the synergistic effects would be mediated by increased DNA damage and subsequent tumor cell apoptosis. Combinatorial effects of KML001 were observed not only in chemo- and radiosensitive glioblastoma cell line, U87MG, but also in the resistant cell line, U251MG. In the U87MG glioblastoma xenograft models, KML001 did not have systemic toxicity but showed synergistic therapeutic effects in combination with temozolomide or irradiation to reduce tumor volumes significantly. These data indicated that KML001 could be a candidate sensitizer to potentiate therapeutic effects of conventional cytotoxic treatment for glioblastoma.

## 1. Introduction

Glioblastoma (GBM) is the most malignant form of primary brain tumors, which shows aggressive cancer cell proliferation, strong invasive capacity into adjacent normal brain tissue, and massive angiogenesis [1]. The current standard therapies for GBM patients are the concomitant fractionated radiotherapy and chemotherapy with DNA methylating agent, temozolomide (TMZ), following surgical removal. Nevertheless, median survival of GBM patients is known to be about 4 months without therapy and 15 months with standard therapies [2].

TMZ has the ability to alkylate/methylate DNA, which mostly occurs at the N7 or O6 position of guanine residues. The methylation leads to DNA strand breaks and triggers apoptotic tumor cell death. Radiation treatment also inducts DNA double strand breakage in cancer cells and thus blocks their ability to proliferate further [3, 4]. However, chemotherapy and radiotherapy have been known to provoke various resistance mechanisms in which DNA repair may play a role [5–9]. The increased DNA repair protein, O^6^-methylguanine-DNA methyltransferase (MGMT), is correlated with TMZ resistance in GBM [10, 11]. Loss of MSH6, a DNA mismatch repair protein, is also found in the recurrence of temozolomide + ionizing radiation treated GBMs [12]. The disruption of Rad51-mediated homology-directed repair (HDR) or prevention of G2 checkpoint activation selectively sensitized GBM cells to radiation [13]. Therefore, the resistance to these TMZ and radiation therapies might be overcome through additional irreparable DNA damage.

Various combination strategies to overcome radiotherapy and/or TMZ chemotherapy resistance in GBM have been examined [14–16]. Some of arsenic compounds, especially, that have been used as effective chemotherapeutic agents in various solid tumors could be viable candidates [17–19]. Among them, sodium meta-arsenite, KML001, is a telomere-targeting agent, which has entered phase I clinical trials of advanced non-small cell lung cancer and other platinum responsive malignancies in ClinicalTrials.gov. KML001 directly targets telomeres of chromosomes and then provokes activation of the DNA damage signaling and rapid telomere erosion in prostate cancer cells [20]. In addition, this agent has shown synergistic antitumor activities at combination treatment with irinotecan in various tumor models [21–23]. Since KML001 enhances DNA damage and cell apoptosis, the resistance to TMZ chemotherapy and radiotherapy of GBM could be overcome through additional DNA damage by KML001.

Here, we reported the synergistic efficacy of a telomere targeting agent, KML001, combined with TMZ or radiation, which provided a candidate solution for unmet needs of conventional therapies for GBM.

## 2. Materials and Methods

### 2.1. Cell Culture and Reagents

The human GBM cell lines U251MG and U138MG (American Type Culture Collection, ATCC, Manassas) were cultured in Dulbecco's modified essential medium (DMEM) and U87MG and U373MG (American Type Culture Collection, ATCC, Manassas) were cultured in Eagle's minimal essential medium (MEM). All mediums were supplemented with 10% fetal bovine serum (FBS, Gibco, USA), penicillin (100 units/mL, Gibco), and streptomycin (100 *μ*g/mL, Gibco). These cells were maintained at 37°C in an incubator flushed continuously with 5% CO_2_.

KML001 (Sodium meta-arsenite) was purchased from Sigma-Aldrich (USA) and 100 mmol/L stock solutions were prepared in 1X phosphate buffered saline (PBS, Gibco). Working concentrations were freshly prepared daily by diluting the stock with PBS. Temozolomide was purchased by Sigma-Aldrich and dissolved in 10% dimethyl sulphoxide (DMSO, Sigma-Aldrich).

### 2.2. Colony Formation Assay

U251MG, U373MG, and U138MG (100 cells/2 mL medium) were seeded in 6-well plates and U87MG was seeded in 100 mm culture dishes (1,000 cells/10 mL medium). After 24 hours, cells were treated with KML001 (0.01, 0.01, 4, 8, 10, and 100 *μ*M), temozolomide (10 and 20 *μ*M), or Irradiation (1, 2, 3, and 4 Gy). After incubation for 10 days, all cells were fixed with 100% methanol and stained with 0.01% or 0.125% crystal violet (Sigma-Aldrich). Colonies containing more than 50 cells were counted as a representative of clonogenic cells. The survival fraction was calculated using the following formula: ((number of colonies formed after treatment)/(number of cells seeded × plating efficiency)), where plating efficiency is the number of colonies to the number of seeded cells [24].

### 2.3. Western Blotting Analysis

Cells were treated with KML001 (5 or 10 *μ*M) or temozolomide (100, 200 or 400 *μ*M) or Irradiation (3, 6 or 9 Gy) for 48 hours. All cells were lysed in NP40 lysis buffer (50 mM Tris, pH 7.4, 250 mM NaCl, 5 mM EDTA, 50 mM NaF, 1 mM Na_3_VO_4_, 1% Nonidet P40, 0.02% NaN_3_) adding protease inhibitor cocktail tablets (Sigma-Aldrich) and phenylmethanesulfonyl fluoride (PMSF, Sigma-Aldrich). After quantitative analysis, the equal amounts of proteins were used for western blotting. Apoptotic pathway proteins were confirmed using rabbit monoclonal cleaved PARP antibody (1 : 1,000; Cell Signaling Technology, USA) and rabbit monoclonal caspase-3 antibody (1 : 1,000; Cell Signaling Technology). Loading control was used mouse monoclonal *β*-actin antibody (1 : 5,000; Santa Cruz Biotechnology, USA). Antibodies were visualized with horseradish peroxidase-conjugated secondary antibodies (Santa Cruz Biotechnology) and the Amersham ECL Prime Western Blotting Detection Reagent (GE Healthcare, UK).

### 2.4. Immunocytochemistry

For immunocytochemistry (ICC) analysis, both U251MG and U87MG cells (3 × 10^3^ cells/well) were cultured on Nunc Lab-Tek II Chamber Slide System (Thermo Scientific, USA). The cells were washed three times with cold 1X PBS and fixed by 4% paraformaldehyde for 10 minutes. The fixed cells were permeabilizated in 0.5% Triton X-100 for 10 minutes and blocked with 1% bovine serum albumin (BSA, Santa Cruz Biotechnology) for 1 hour at room temperature and then incubated with primary antibodies *γ*-H2AX (Upstate/Millipore, USA) at room temperature for 1 hour. Continuously, the cells were incubated with Alexa-flour 488 dye-conjugated secondary antibodies (Invitrogen, USA) for 1 hour at room temperature. These cells were stained with 4′, 6-diamidino-2-phenylindole (DAPI, Invitrogen) for nuclear detection and viewed using a confocal laser scanning microscopy (Carl-Zeiss, Germany).

### 2.5. Xenograft Tumor Model

For efficacy test of KML001, temozolomide and Irradiation* in vivo*, we established the orthotopic xenograft models by intracranial injection using 6-week-old female athymic nude mice. To establish the orthotopic xenograft models, U87MG cells (2 × 10^5^/5 *μ*L Hank's balanced salt solution (HBSS), Gibco) were stereotypically injected into the left striata of mice (coordinate; AP +0.5, ML +1.7, DV −3.2 mm from Bregma). Each group had eight mice, and mice were medicated by KML001 (5 mg/kg for every day via per oral) at 1 day after tumor cells injection. Temozolomide (2 mg/kg × 5 via per oral) and whole brain irradiation (2 Gy × 5) exposed at 18 days to 22 days after tumor cells injection. All animals were sacrificed at 28 days, median survival of the orthotopic xenograft model using U87MG cells. Tumor diameter was measured using vernier caliper and tumor volume determined by calculating the volume of an ellipsoid using the formula: (length × (width)^2^ × 0.5). After mice sacrifice, the data of tumor diameter measurement were excluded in the case that cells had leaked through to cerebral ventricles.

### 2.6. Statistics Analysis

The results were expressed as mean values ± standard deviation (S.D) or standard effort (S.E). Statistical comparisons were analyzed by one-way analysis of variance (ANOVA) followed by the least significant difference (LSD) test. A significant level of *P* < 0.05 was used for all tests. SPSS-PASW statics software version 18.0 was used for all the statistical analyses.

## 3. Results and Discussion

### 3.1. Survival of GBM Cells Was Significantly Inhibited by Combination Treatments with KML001 and TMZ or Irradiation

We performed cell colony formation assay to determine whether the combination treatments with KML001 and TMZ or irradiation decreased GBM cell survival and increased drug sensitivity. This assay is an* in vitro* clonogenicity test based on the ability of a single cell to grow into a colony; it permits evaluation of the oncogenic potential of a single cell [25]. In the colony formation assay, numbers of colonies rather than total cell numbers were compared. Therefore, proliferation rate would make little effects on the results of the assay, although GBM cell lines showed different proliferation rates (Supplementary Figure 1 in Supplementary Material available online at http://dx.doi.org/10.1155/2014/747415). Moreover, differential cell proliferation prior to the treatments would not affect the results since GBM cells hardly proliferated within 24 hours after seeding (Supplementary Figure 1).

**Figure 1:**
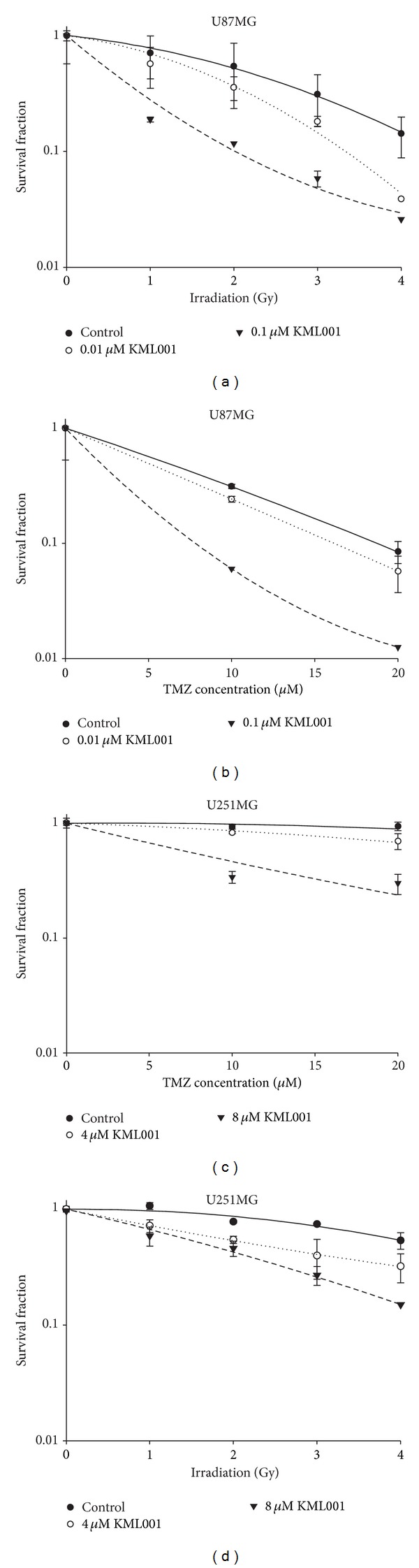
Clonogenic survival of GBM cells was significantly decreased by the combination of KML001 and TMZ or irradiation. GBM cells were seeded at 100/dish density in culture dishes and incubated for 10 days with indicated treatment. Survival fraction was demonstrated. (a) U251MG, combination with KML001 and TMZ, (b) U87MG, combination with KML001 and TMZ, (c) U251MG, combination with KML001 and irradiation, and (d) U87MG, combination with KML001 and irradiation.

**Figure 2:**
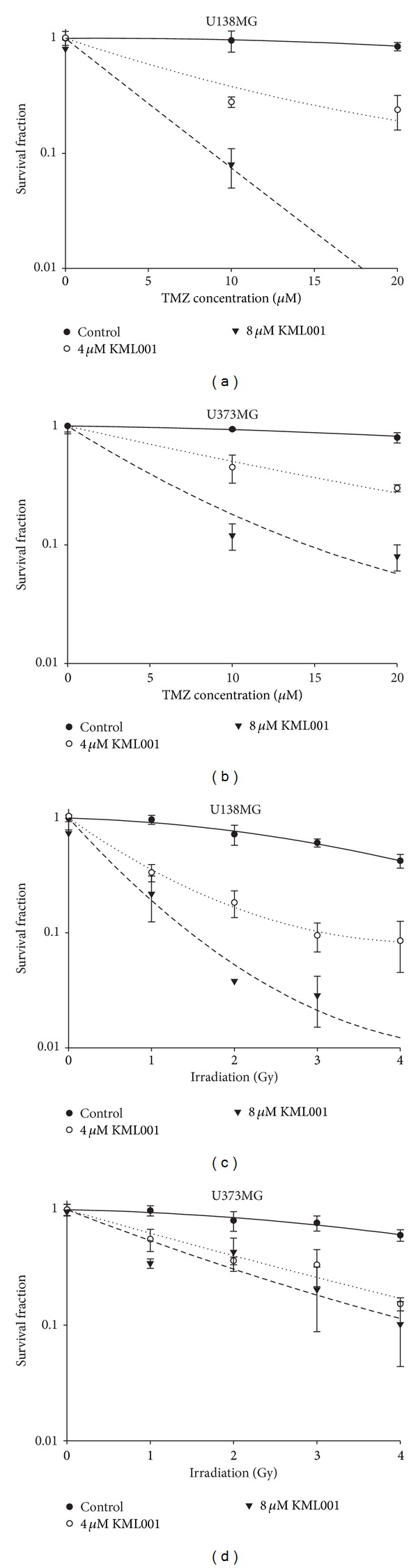
Clonogenic survival of GBM cells was also significantly decreased by the combination with KML001 and TMZ or irradiation in various GBM cells. GBM cells were seeded at 100/dish density in culture dishes and incubated for 10 days with indicated treatment. Survival fraction was demonstrated. (a) U373MG, combination with KML001 and TMZ, (b) U138MG, combination with KML001 and TMZ, (c) U373MG, combination with KML001 and irradiation, and (d) U138MG, combination with KML001 and irradiation.

As a result, we identified that KML001 significantly increased sensitivities of both U251MG and U87MG GBM cells to TMZ and irradiation in a dose dependent manner (Figure 1). In U251MG GBM cells, 8 *μ*M KML001 decreased cell survival 3.8-fold at 20 *μ*M TMZ and 3.6-fold at 4 Gy irradiation (Figures 1(a) and 1(c)). In U87MG GBM cells, 0.1 *μ*M KML001 decreased cell survival 6.7-fold at 20 *μ*M TMZ and 5.0-fold at 4 Gy irradiation (Figures 1(b) and 1(d)). Other GBM cell lines such as U373MG (Figures 2(a) and 2(c)) and U138MG (Figures 2(b) and 2(d)) also showed similar synergistic effects of KML001.

GBM is one of the most resistant tumors to conventional cytotoxic therapies, which results in minimal survival benefit from standard chemotherapy and radiotherapy. To overcome resistance, current studies concentrate on combinations with other agents and on development of novel molecular-targeting agents [14–16]. Previously, the anticancer efficacy of KML001 in combination with irinotecan was evaluated by Moon et al. [21]. They showed that vascular disrupting properties and DNA damage effects at the telomeres of chromosome of KML001 were involved in the enhanced anticancer activity of irinotecan [21]. We also observed that KML001 significantly enhanced* in vitro *sensitivity of TMZ and irradiation in 4 independent GBM cell lines. These results suggested that KML001 could acts as a chemo- and radiosensitizer in GBM.

On the other hand, we also recognized that U87MG cell line showed sensitive response to both TMZ and irradiation, while the U251MG cell line was resistant. Therefore, we further evaluated the differential changes in DNA damage and apoptosis signaling* in vitro* and tumor growth of chemo- and radioresistant U251MG cells, compared with those of chemo- and radiosensitive U87MG cells,* in vivo*.

### 3.2. DNA Damage and Cell Apoptosis Induced by TMZ or Irradiation Were Enhanced by KML001

TMZ and irradiation lead to cancer cell apoptosis through DNA mismatch-repair [10, 11] and DNA double strand break (DSB) events [3]. Previously, many studies focused on the explanation for mechanisms of the phosphorylated *γ*-H2AX in DNA damage signaling and repair [26–28]. Notably examination of *γ*-H2AX foci formation is a powerful tool to measure DNA DSB formation and cellular response to genomic damage [28]. In addition, caspase-3 is frequently activated by death protease to catalyze the specific cleavage of many key cellular apoptosis-inducing proteins including PARP [29, 30]. Therefore, to elucidate the combination mechanism of KML001 in GBM cells, we analyzed phosphorylated *γ*-H2AX formation and caspase-3/PARP cleavage in response to the combination treatments.

**Figure 3:**
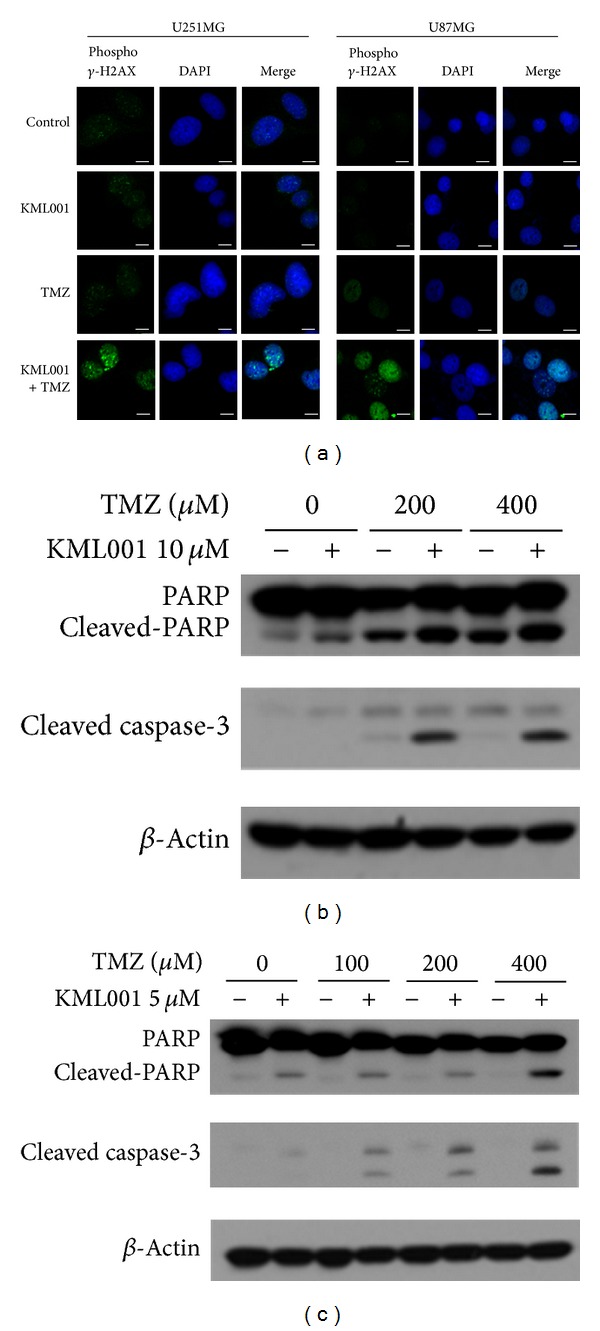
DNA damage and apoptosis were enhanced by* in vitro* combination treatment of KML001 and TMZ. (a) Phosphorylated *γ*-H2AX was detected by immunocytochemistry after 40 minutes treatment with 200 *μ*M TMZ and KML001 [10 *μ*M (for U251MG) or 5 *μ*M (for U87MG)]. DAPI (blue) = nuclei. (b, c) U251MG (b) and U87MG (c) cells were treated* in vitro* as indicated for 48 hours. Western blot analysis for PARP, cleaved PARP, or cleaved caspase-3 was performed. *β*-actin = loading control.

Phosphorylated *γ*-H2AX a DNA DSB marker was detected in the KML001 and TMZ combination group in both TMZ-resistant U251MG and sensitive U87MG cell lines, while the KML001 or TMZ single treatments showed little phosphorylated *γ*-H2AX by immunocytochemistry (Figure 3(a)). Moreover, 10 *μ*M KML001 combination dramatically enhanced the protein expression of cleaved PARP and caspase-3 at 200 and 400 *μ*M TMZ treatment in TMZ-resistant U251MG cells (Figure 3(b)). In TMZ-sensitive U87MG cells, cleaved PARP and caspase-3 were hardly observed by TMZ singe treatment. In contrast, 5 *μ*M KML001 combined with 400 *μ*M TMZ strikingly increased cleaved PARP level, while TMZ provoked a dose-dependent increase in cleaved caspase-3 upon combination with 5 *μ*M KML001 (Figure 3(c)).

**Figure 4:**
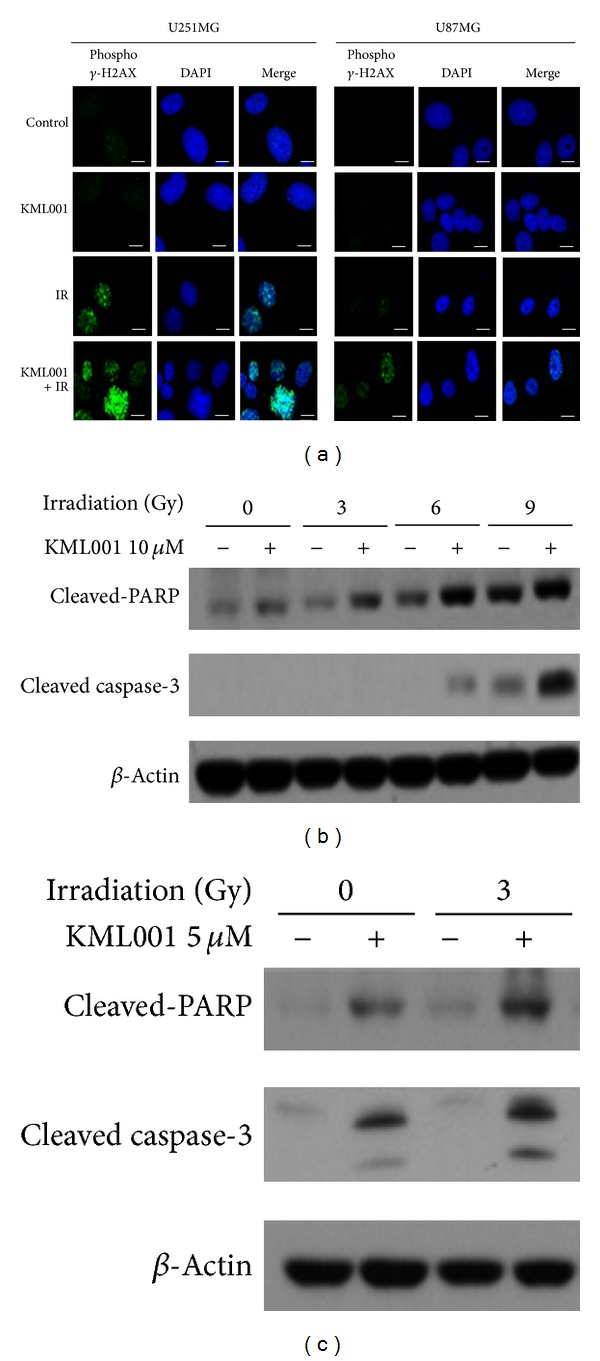
DNA damage and apoptosis were enhanced by the* in vitro* combination treatment of KML001 and irradiation. (a) Phosphorylated *γ*-H2AX was detected by immunocytochemistry after 40 minutes treatment with 3 Gy irradiation and KML001 10 *μ*M (for U251MG) or 5 *μ*M (for U87MG). DAPI (blue) = nuclei. (b, c) U251MG (b) and U87MG (c) cells were treated* in vitro* as indicated for 48 hours. Western blot analysis against PARP, cleaved PARP, or cleaved caspase-3 was performed. *β*-actin = loading control.

Next, we performed the same experiments with the combination of KML001 with irradiation. In both radiation-resistance U251 MG and sensitive U87MG, the phosphorylated *γ*-H2AX was dramatically increased by the combination treatment of KML001 and 3 Gy irradiation, compared with the control or single treatments (Figure 4(a)). In common with the combination treatment with KML001 and TMZ, KML001 increased the protein expression of the apoptotic marker proteins, cleaved PARP and caspase-3 at 3 Gy irradiation in U251MG cells (Figure 4(b)). In U87MG cells, KML001 also dramatically potentiated the irradiation dose-dependent increase in cleaved PARP and caspase-3 (Figure 4(c)).

Arsenic based pharmaceuticals have been reported to inhibit viability of pancreatic cancer stem cells [22]. Accordingly, the combination treatments with low dose gemcitabine synergistically inhibited tumorigenesis of pancreatic xenograft model [22]. The combination treatment with arsenic compounds and cytotoxic agents synergistically enhanced DNA damage and cell apoptosis [23]. Recently, trivalent arsenical KML001 was also reported to induce the ROS-associated DNA damage* via* direct biding to telomeric sequences in prostate cancer cells [20]. Binding with telomeric sequences is enhanced when cells have short telomeres, which results in cancer cell specific effects of KML001; cancer cells have shorter telomeres than somatic cells such as astrocytes [20, 31]. Although the activity and/or mutation status of telomerase reverse transcriptase (TERT) could make influences on the length of telomere, KML001 has little effect on telomerase activity [20]. Moreover, KML001 provoked apoptosis of TERT-low leukemia cells [32]. Therefore, cytotoxic activities of KML001 would be directly affected by the telomere length, not by the mutation status, expression, and/or activities of TERT in cancer cells.

To conclude in this study, we identified that KML001 combined with TMZ or irradiation potentiated DNA damage and subsequent GBM cell apoptosis. Since KML001 induced therapeutic sensitivity to TMZ and irradiation in the chemo- and radioresistant cell, U251MG, as well as the chemo- and radiosensitive cell, U87MG, KML001 would have broad therapeutic indications for GBM.

### 3.3. KML001 Combined with TMZ or Irradiation Synergistically Decreased Tumor Volume in U87MG GBM Orthotopic Xenograft Models

**Figure 5:**
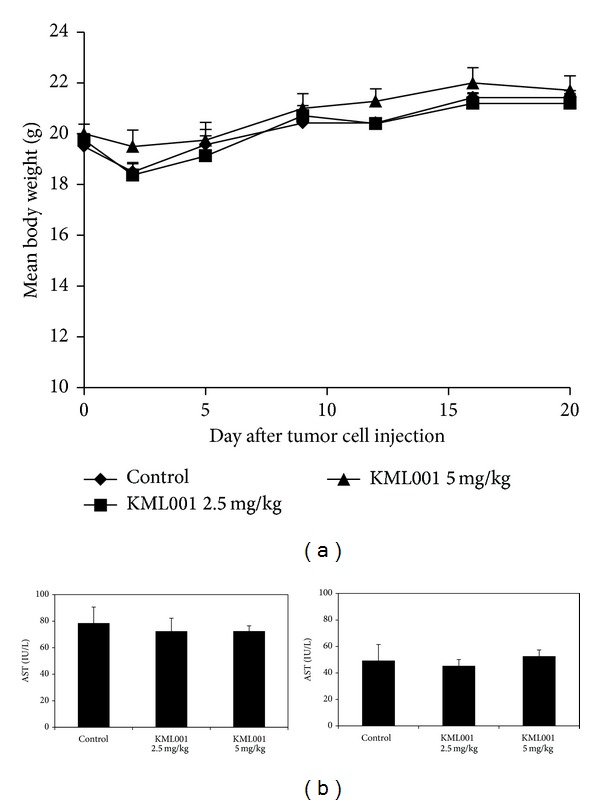
KML001 showed little* in vivo* systemic toxicity in the U87MG GBM orthotopic xenograft models. KML001 (2.5 or 5 mg/kg) was orally administrated into U87MG orthotopic xenograft models every day from 1 day after tumor cell implantation. (a) Body weight was measured twice a week until 20 days after tumor cell implantation. (b) The level of AST and ALT was analyzed in mice serum on the 20th day after tumor cell implantation.

We employed U87MG orthotopic xenograft models to confirm the* in vitro* combinational treatment result of KML001,* in vivo*. Firstly, we tested whether KML001 had* in vivo *toxicity by measuring body weight and liver enzyme (AST and ALT) level changes in the orthotopic model. KML001 treatment resulted in no change in body weight nor in levels of AST and ALT, validating low* in vivo* toxicity (Figure 5).

**Figure 6:**
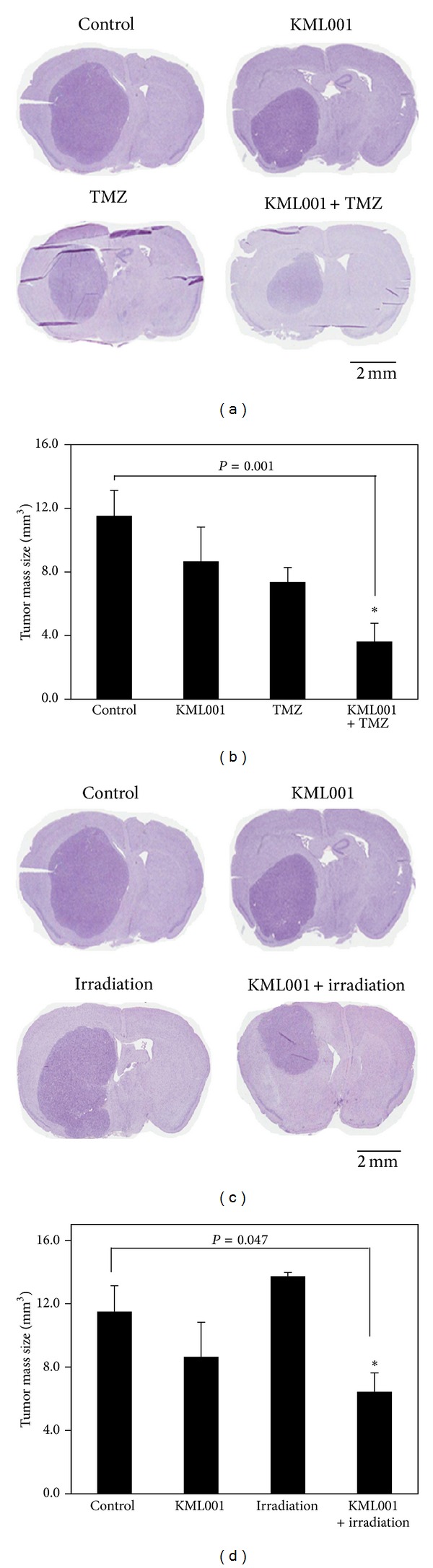
Tumor volume of U87MG xenograft tumors was significantly reduced by the combination treatment with KML001 and TMZ or irradiation. The orthotopic xenograft models were treated with normal saline (control, every day from 1 day after tumor cell implantation), KML001 (5 mg/kg, every day from 1 day after tumor cell implantation), TMZ (2 mg/kg, 5 times daily from 18th to 22nd day after tumor cell implantation), irradiation (2 Gy, 5 times daily from 18th to 22nd day after tumor cell implantation), or KML001 + TMZ or irradiation. Tumor volume (length × (width)^2^  × 0.5) was analyzed on the 28th day after tumor cell implantation. (b and d) Data represent mean ± standard deviation. *P < 0.05.

The U87MG orthotopic xenograft models were treated with (1) control (normal saline, every day from 1 day after tumor cell implantation), (2) KML001 (5 mg/kg, every day from 1 day after tumor cell implantation), (3) TMZ (2 mg/kg, 5 times daily from 18th to 22nd day after tumor cell implantation) or irradiation (2 Gy, 5 times daily from 18th to 22nd day after tumor cell implantation), or (4) KML001 + TMZ or irradiation. Tumor volume was analyzed on the 28th day after tumor cell implantation. The combination treatment with KML001 and TMZ significantly decreased xenograft tumor volume, while the single treatments of KML001 or TMZ slightly decreased tumors size (Figure 6(a)). The combination treatment with KML001 and TMZ decreased tumor volume 2.4- and 2.0-fold compared with KML001 and TMZ single therapy, respectively (Figure 6(b)). Similarly, the combination treatment with KML001 and irradiation reduced tumor volume significantly (Figure 6(c)). Although irradiation showed no effects (Figure 6(c) bottom and left),tumor volume decreased 1.8-fold by the combination therapy compared with KML001 single treatment (Figure 6(d)).

According to a previous report, the treatment with TMZ caused a substantial growth delay of U87MG xenografts tumor, while irradiation did not affect tumor growth [33]. Here, we also confirmed that TMZ had higher anticancer effects than irradiation in U87MG xenografts. Furthermore, we identified that KML001 not only potentiated antitumor effects of TMZ against U87MG xenograft tumors but also reversed* in vivo* resistance of U87MG cells to irradiation. Although tumor volume decreased by single treatments of TMZ or KML001, they were not statically significant. Only xenograft tumors were significantly reduced by the combination treatment with KML001 and TMZ (*P* = 0.001) or irradiation (*P* = 0.047) compared with the nontreatment group.

## 4. Conclusions

In this study, we demonstrated that KML001 potentiated antitumor effects of conventional cytotoxic treatment for GBM: TMZ chemotherapy and radiotherapy* in vitro* and* in vivo*. These synergistic effects could be mediated by increased DNA damage, which would further provoke GBM cell apoptosis. Since KML001 alone did not show any* in vivo* systemic toxicities, KML001 could be a viable candidate for a combinational sensitizer in GBM treatment.

## Supplementary Material


*In vitro* proliferation of GBM cell lines. GBM cells were seeded at 1000/well density in 96-well culture plates, and incubated for 0, 24, or 48 hours (n=6 for each group). Cell numbers were determined by EZ-cytox cell viability kit (DAEIL Lab) according to the manufacturer's protocol..
