# Supplementary material for: KML001, a Telomere-Targeting Drug, Sensitizes Glioblastoma Cells to Temozolomide Chemotherapy and Radiotherapy through DNA Damage and Apoptosis
Source: Biomed Res Int. 2014 Sep 10;2014:747415. doi: 10.1155/2014/747415 (PMC4176651; doi:10.1155/2014/747415)
Supplement: Supplementary file 1 — In vitro proliferation of GBM cell lines. GBM cells were seeded at 1000/well density in 96-well culture plates, and incubated for 0, 24, or 48 hours (n=6 for each group). Cell numbers were determined by EZ-cytox cell viability kit (DAEIL Lab) according to the manufacturer's protocol.. [file 747415.f1.docx]

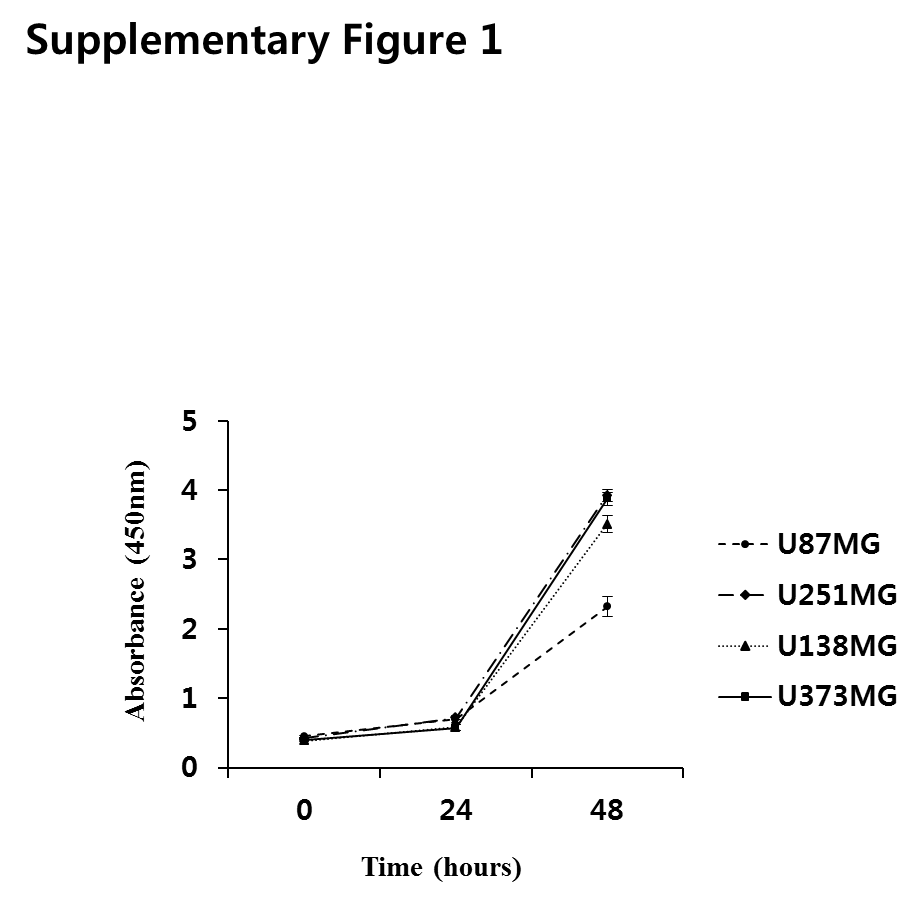


**Supplementary Figure 1. *In vitro* proliferation of GBM cell lines.** GBM cells were seeded at 1000/well density in 96-well culture plates, and incubated for 0, 24, or 48 hours (n=6 for each group). Cell numbers were determined by EZ-cytox cell viability kit (DAEIL Lab) according to the manufacturer’s protocol.
